# Synthesis of Volumetric Ring Antenna Array for Terrestrial Coverage Pattern

**DOI:** 10.1155/2014/260949

**Published:** 2014-02-18

**Authors:** Alberto Reyna, Marco A. Panduro, Carlos Del Rio Bocio

**Affiliations:** ^1^Unidad Académica Multidisciplinaria Reynosa-Rodhe, Universidad Autónoma de Tamaulipas (UAT) Carretera Reynosa-San Fernando, 88779, Reynosa, TAMPS, Mexico; ^2^Universidad Pública de Navarra, Campus de Arrosadia, Los Tejos, 31006 Pamplona, Spain

## Abstract

This paper presents a synthesis of a volumetric ring antenna array for a terrestrial coverage pattern. This synthesis regards the spacing among the rings on the planes *X*-*Y*, the positions of the rings on the plane *X*-*Z*, and uniform and concentric excitations. The optimization is carried out by implementing the particle swarm optimization. The synthesis is compared with previous designs by resulting with proper performance of this geometry to provide an accurate coverage to be applied in satellite applications with a maximum reduction of the antenna hardware as well as the side lobe level reduction.

## 1. Introduction

In satellite applications, there exist many services such as navigation, aeronautical communications, weather forecast, and so forth that require preferably satellites for uniform terrestrial coverage usually called isoflux radiation [1]. A critical factor for these satellites is the power efficiency consumed over the whole onboard antenna system and its volume occupation. Commonly, reflectors as well as active lenses are utilized for different satellites systems where the volume occupation and weight imply difficulties for the accommodation onboard the satellite [2–5]. Since any problem with the movement could block the full satellite system, introducing mechanical movement to the antenna system to refocus the beam is not a real option. A proper alternative is the use of antenna arrays with reduction of hardware as much as possible while avoiding the degradation of the good performance. The use of antenna arrays mounted in satellites is another alternative to provide required characteristics of radiation in satellites. In fact, in recent years, many antenna arrays have been employed in satellites. But surely, the spatial uniform distribution of the antenna elements in antenna arrays increases the size of the beamforming network whereas many levels of excitations complicate the heat dissipation system in the satellite; that is, having many amplifiers with no uniform gains could complicate the heat dissipation and even could fuse the antenna system. That is why the antenna community has recently made efforts to design antenna arrays for satellites by using uniform excitations so that the beamforming becomes simpler. Particularly, the state of art of antenna arrays for a uniform terrestrial coverage includes some deterministic approaches for synthesizing the spatial uniform arrays [6, 7] and spatial aperiodic arrays [8]. Furthermore, in order to satisfy even more the above proper results, recently, the evolutionary and swarm algorithms have been utilized to synthesize antenna arrays for a global isoflux radiation in satellites with a considerable reduction of the antenna excitations. These results revealed the effectiveness of the evolutionary and swarm algorithms to design aperiodic arrays [9] and concentric ring arrays [10, 11]. Although a considerable simplification of the antenna hardware by means of synthesis of two-dimensional arrays was achieved, three-dimensional antenna array is not even taken into consideration for a uniform terrestrial coverage. In fact, three-dimensional antenna arrays have been only synthesized [12] and even manufactured for pencil beams in satellites applications [13, 14]. Also, even active lens under a volumetric topology has been recently manufactured for only pencil beams in satellites applications [15]. A preliminary study of three-dimensional antenna arrays in order to provide uniform global coverage has been presented in [16]. That is, the inspiration to contribute with this new paper focused on a study of the behavior of three-dimensional antenna arrays to provide a uniform global coverage with an isoflux shape pattern. This paper offers novel contributions beyond those of the previous work.

This paper presents the analysis and evaluation of a three-dimensional antenna array which we name as volumetric ring array (VRA) for a terrestrial coverage pattern. This synthesis regards the spacing among the rings on the planes *X*-*Y* and the positions of the rings in the *z*-direction. The optimization is carried out by implementing the particle swarm optimization (PSO) [17, 18], and the next study cases are considered: (a) a VRA for GEO (geostationary earth orbit) satellites in the case of uniform excitation and the case of concentric excitation, (b) a VRA for MEO (medium earth orbit) satellites in the case of uniform excitation and the case of concentric excitation, and (c) a VRA for LEO (low earth orbit) satellites by considering concentric excitation. Furthermore, this paper illustrates a comparative evaluation between the case of uniform excitation and the case of concentric excitation. The synthesis is compared with previous designs by resulting with proper performance of this geometry to provide an accurate coverage to be applied in satellite application with a maximum reduction of the antenna hardware as well as the side lobe level reduction.

## 2. Volumetric Ring Array Model

**Figure 1:**
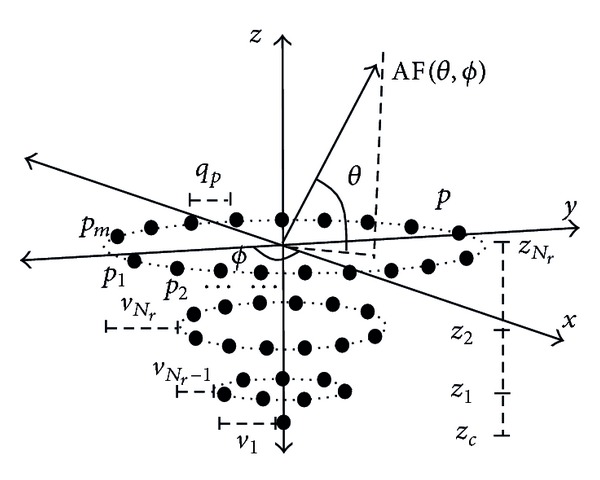
Volumetric ring antenna array.

Assume a radial geometry with a VRA shown in Figure 1 of *N*_*T*_ elements spaced on the plane *X*-*Y* is given by

(1) $$\begin{array}{c} N_{T}=1+\overset{N_{r}}{\underset{p=1}{\sum }}N_{p} \end{array}$$

The array factor for this VRA is now given by

(2) AF(θ,ϕ,L,S,Z)=wc +∑p=1Nr ∑m=1Npwpmejk[rpucos⁡ϕm+rpvsinϕm+zpw],

where *u* = sin*θ*cos⁡*ϕ*, *v* = sin*θ*sin*ϕ*, *w* = cos⁡*θ*, *N*_*r*_ represents the number of the rings, *N*_*p*_ represents the number of elements on the ring *p*, *ϕ*_*m*_ = 2*π*(*m* − 1)/*N*_*p*_ represents the angular position of the element *m* on the ring *p*, *r*_*p*_ is the radius of ring *p*, and *k* = 2*π*/*λ* is the wave constant. The *w*_*c*_ is the amplitude excitation of the central element. The term *w*_*pm*_ is the amplitude excitation of the element *m* on the ring *p* defined in the vector *W* = {*w*_*c*_, *w*_11_, *w*_12_,…, *w*_*N*_*r*_*N*_*p*__}. The levels of amplitudes are defined as *L* = {*l*_1_, *l*_2_,…, *l*_*g*_} where *l*_*g*_ is the excitation value for the elements in a group of rings of the array. The radius of each ring defines the spacing among the rings in the next way *s*_1_ = *r*_1_, *s*_2_ = (*r*_2_ − *r*_1_), *s*_3_ = (*r*_3_ − *r*_2_),…, *s*_*N*_*r*__ = (*r*_*N*_*r*__ − *r*_*N*_*r*−1__) which are arranged in the set of real numbers *S* = {*s*_1_, *s*_2_,…, *s*_*N*_*r*=*p*__}. In addition, the position *z*_*p*_ represents the position of the ring *p* which is defined in the set of real numbers *Z* = {*z*_*c*_, *z*_1_, *z*_2_,…, *z*_*N*_*r*__}, where *z*_*c*_ is the position of the central element and *z*_*N*_*r*__ is the position of the bigger ring. For this VRA, the spacing among the antenna elements in the ring *p* is *q*_*p*_ = 2*πr*_*p*_/*N*_*p*_. In the case of *s*_1_ = *s*_2_ = ⋯ = *s*_*N*_*r*__ = *s*_max⁡_, the maximum aperture might become *A*_max⁡_ = *N*_*r*_ *s*_max⁡_, where *s*_max⁡_ is the boundary in the search space of the elements in the vector *S*.

## 3. Problem Statement

### 3.1. Isoflux Requirements and Fitness Function

**Figure 2:**
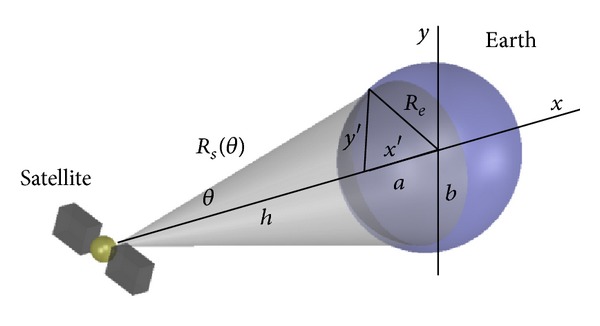
Electromagnetic illumination in the equatorial pole of the earth.

In order to shape radiation with no variation in the strength power density to any point of the illuminated earth surface, as seen in Figure 2, we consider an elliptical shape of the earth to deeply study the behavior of the antenna arrays for an isoflux radiation in order to mimic the real shape of the earth as accurately as possible. Since this framework, an accurate radiation pattern can be calculated as a function of *R*(*θ*) in the coverage area as follows [11]:

(3) R2(θ)(sin2θb2+cos⁡2θa2)  +R(−2(e+a)cos⁡θa2)+((e+a)2a2−1)=0,

where *R*(*θ*) indicates the relative distance of the satellite to any point of the illuminated earth surface, *e* represents the height of the satellite, *a* is the equatorial radius of the earth, and *b* is the polar radius of the earth. Assuming any sweep in the azimuth plane, the function *R*(*θ*) should be identical according to the elliptical symmetric shape of the earth. The angular position for the edge of coverage (EOC) is determined by the maximum value of the *R*(*θ*). The fitness function of this design problem is formulated as follows:

(4) of=|AF(θr,ϕ,L,S,Z)−R(θr)|2 +|AF(θSLL,ϕSLL,L,S,Z)max⁡(AF(θ,ϕ,L,S,Z))|,

where *θ*_*r*_ is the range of the elevation plane for the coverage area and (*θ*_SLL_, *ϕ*_SLL_) is the angle where the maximum side lobe level is attained. The optimization is to minimize two aspects: firstly, the sum of the square error for each angle in the field of view (FOV) zone between the real-elliptical shape of the earth and the array factor and secondly, the maximum side lobe level. The first term of the fitness function is the mean squared error between the module of the array factor AF(*θ*_*r*_, *ϕ*) and the prescribed pattern *R*(*θ*_*r*_) for all the cuts of *ϕ*. The array factor AF(*θ*_*r*_, *ϕ*) is taking just in the half of the azimuth plane *ϕ*, that is, [0, *π*], and in the range of the elevation plane *θ* of [0, *θ*_*r*_]. The prescribed pattern *R*(*θ*_*r*_) is also taking just the half of the curvature [0, *θ*_*r*_] which is sweeping with the same distribution for each value of *ϕ* in AF(*θ*_*r*_, *ϕ*). The reduction of the side lobe level is recommended because it would not be good to lose a great amount of energy in the angular range that is not illuminating the Earth. In addition, it could be noted that this lost energy might even interfere with other satellites and so forth. As a matter in fact, if we used certain type of antenna element for any implementation in order to cancel the side lobes, it would not ensure that the cancelled energy is being retrieved into the angular range of the cuvature of isoflux shape this energy would be wasted anyway but not in the curvature of the isoflux shape.

The fitness function was properly adjusted by each term with a trial and error method. The term of the error between the prescribed pattern and the isoflux radiation was weighted with a unit coefficient and the term of the side lobe level was weighted by 20 as coefficient.

### 3.2. Particle Swarm Optimization

In this work, the PSO is utilized to synthesize the VRA to achieve the desirable isoflux shape pattern. Each particle is represented by optimization variables. Then, the particles move influenced by its current position, its memory, and the cooperation or social knowledge of the swarm, using only one operator, the so-called velocity operator. Let us suppose a swarm of *H* particles, in which each particle *J*_*H*_ = (*j*_*h*1_,…, *j*_*hD*_) representing a potential solution is defined as a point in *D*-dimensional space. The limits of the parameters *j*_*hd*_ to be optimized define the search space in *D*-dimensions. Iteratively, each particle *h* within the swarm flies over the solution space to a new position *J*_*H*_ with a velocity *V*_*h*_ = (*v*_*h*1_,…, *v*_*hD*_), both updated along each dimension *d*, by the following:

(5) vhd=iw·vhd+c1r1(pbesth,d−xhd)+c2r2(gbestd−jhd),vh≤vd,max⁡  ∀d,jhd=jhd+vhdΔt,

where iw is known as the inertial weight and *c*_1_ and *c*_2_ are the acceleration constants and they determine how much the particle is influenced by its best location (usually referred to as memory, nostalgia, or self-knowledge) and by the best position ever found by the swarm (often called shared information, cooperation or social knowledge), respectively. Moreover, *r*_1_ and *r*_2_ represent two separate calls to a random number function *U*[0, 1], *v*_*d*,max⁡_ is the maximum allowed velocity for each particle used as a constraint to control the exploration ability of the swarm and usually set to the dynamic range of each dimension [19], and Δ*t* is a time step usually chosen to be one. In PSO, the population size, the inertial weight, and the acceleration constants summarize the parameters to be selected and tuned.

## 4. Simulation Results

Three designs of VRA are proposed: (a) *N*_*T*_ = 61 elements in *N*_*r*_ = 4 rings for GEO satellites, (b) *N*_*T*_ = 37 elements in *N*_*r*_ = 3 rings for MEO satellites, and (c) *N*_*T*_ = 19 elements in *N*_*r*_ = 2 rings for LEO satellites. The authors propose this amount of elements in order to obtain the required global coverage. The parameters of the PSO are set as in [8]. Consider a GEO satellite at 36000 km, a MEO satellite at 20000 km, and a LEO satellite at 2000 km of altitude. The prescribed pattern is established in (a) EOC ≈ 9° for GEO, (b) EOC ≈ 14° for MEO, and (c) EOC ≈ 50° for LEO with a suppression of −1.3 dB, −2.1 dB, and −8.6 dB, respectively. For these designs, the aperture is established to be in *S* ∈ [*λ*/2, *λ*] and a reduced range of *Z* ∈ [−1*λ*, 0]. Furthermore, the case of uniform excitation for all antenna elements (*g* = 1) and the case of three levels of concentric excitations (*g* = 3) are studied, that is, one level per each ring as in [8, 9]. The optimization cases were run in MATLAB using a computer with an Intel CPU-i7 860 and 8 gigabytes of memory RAM. The execution time was around 4.5 hours for each optimization.

**Figure 3:**
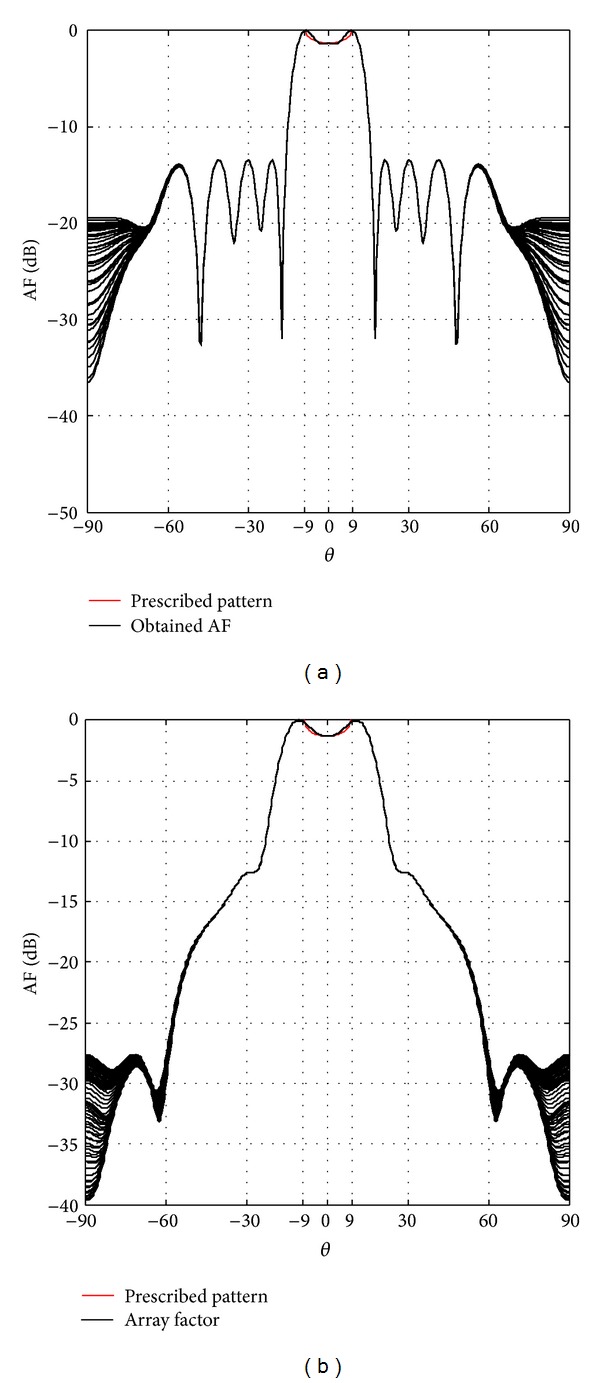
Isoflux radiation for GEO satellites: (a) uniform excitation and (b) concentric excitation.

**Figure 4:**
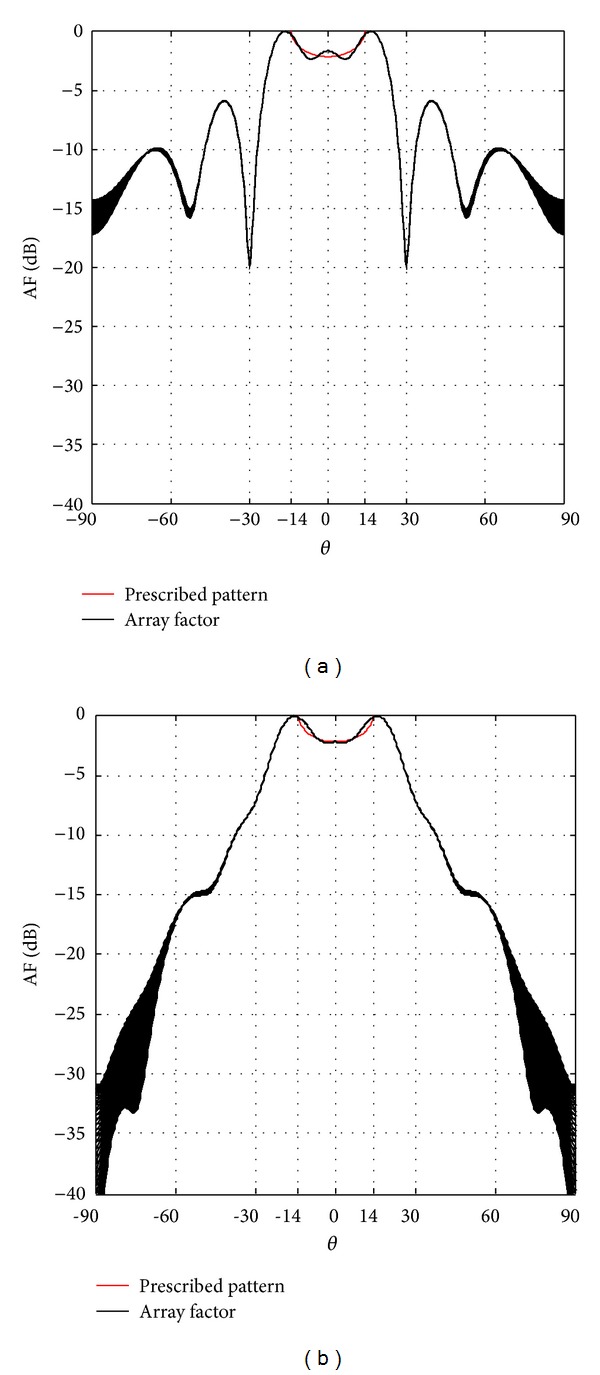
Isoflux radiation for MEO satellites: (a) uniform excitation and (b) concentric excitation.

**Figure 5:**
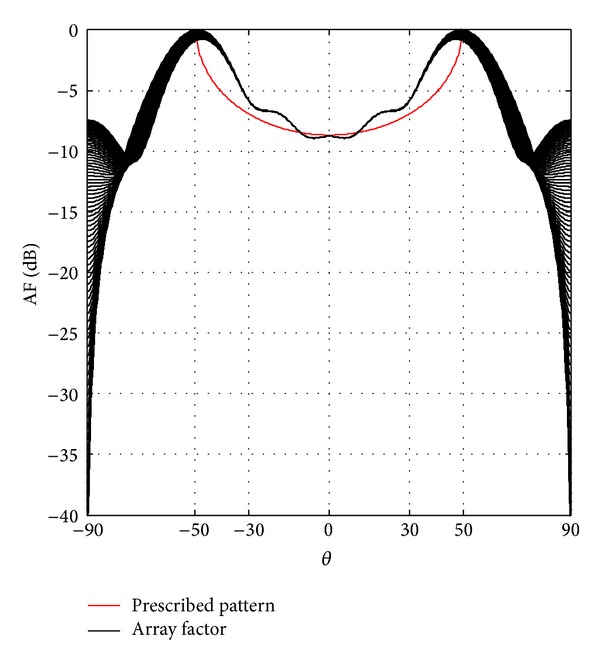
Isoflux radiation for LEO satellites with concentric excitation.

Figure 3 shows the isoflux radiation for a GEO satellite; we can observe the SLL reduction of −SLL ≤ −27 dB for concentric excitations and SLL ≤ −13 dB for uniform excitations.  Figure 4 shows the results for a MEO satellite; in this case a SLL reduction of −SLL ≤ −30 dB for concentric excitations and SLL ≤ −5.8 dB for uniform excitations is obtained. And Figure 5 shows the isoflux radiation for a LEO satellite; the obtained SLL reduction is SLL ≤ −7.4 dB for concentric excitations. It is important to mention that the capability of a VRA for a LEO satellite does not permit an isoflux radiation with uniform excitation due to the wider isoflux curvature. The side lobe level reduction was smaller in the cases of uniform excitations with respect to the cases of concentric excitations. However, note that the case of concentric excitations achieved a shoulder shape in the radiation. This new geometry permits a better accuracy rather than two-dimensional geometries in isoflux shape with the maximum radiation obtained in the EOC. As the directivity of the interest is in the coverage angular range not only in the maximum direction, the maximum discrepancy of this range between the mask and the obtained directivity was obtained. The results in [11, 12] reported a discrepancy of directivity around *|*1 dB*|* and *|*0.5 dB*|* in the isoflux pattern with respect to the prescribed pattern by two-dimensional arrays for GEO and MEO satellites. Now, the VRA permits the possibility of reducing that discrepancy to around *|*0.1 dB*|* which means a greater uniformity in the zone coverage for the cases of GEO and MEO satellites. It is important to state that, by changing the weights to the terms in the fitness function, one could obtain a more directive isoflux radiation but the angular position of the EOC will be affected and the curvature of the isoflux shape will not be as accurate as these VRA designs. This is a trade-off between the accuracy of directivity in the coverage zone and the appearance of shoulders in the radiation. Because of that, the main contribution is the fact that this VRA reduced even more the SLL and the discrepancy of directivity between the prescribed pattern and the obtained array factor with respect to the previous designs presented in [4–9], that is, two-dimensional arrays under a similar aperture. We highlight these results as an alternative to obtain a better accuracy in the isoflux curvature because it was not possible to obtain this accuracy regarding two-dimensional arrays with or without phase excitations. Otherwise, the particular case of a VRA for a LEO satellite has a bigger discrepancy; however, this discrepancy does not affect the uniformity of power density over the earth surface because the power density is exceeding the prescribed pattern in the isoflux curvature and its value is accurate in the nadir direction.

**Figure 6:**
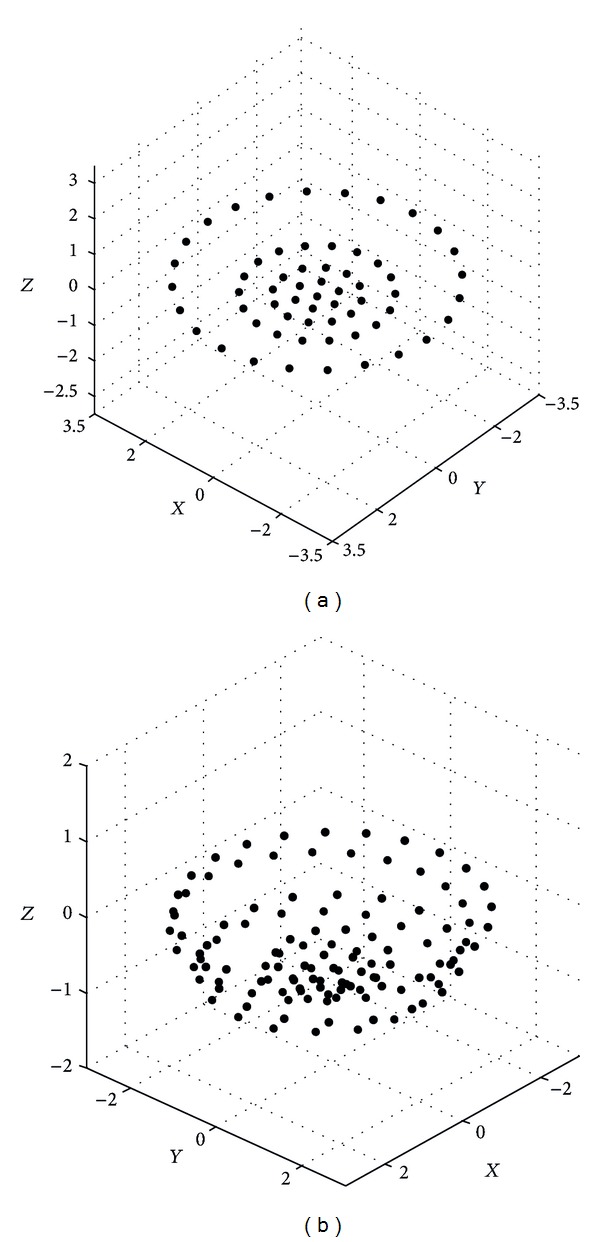
Element distribution of VRA for GEO satellites: (a) uniform excitation and (b) concentric excitation.

**Figure 7:**
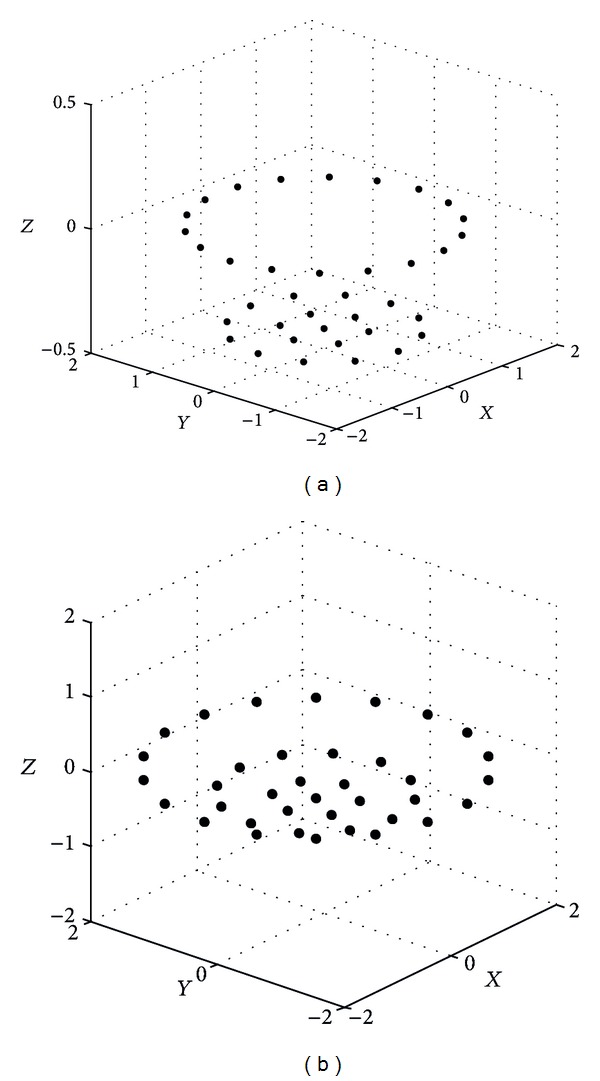
Element distribution of VRA for MEO satellites: (a) uniform excitation and (b) concentric excitation.

**Figure 8:**
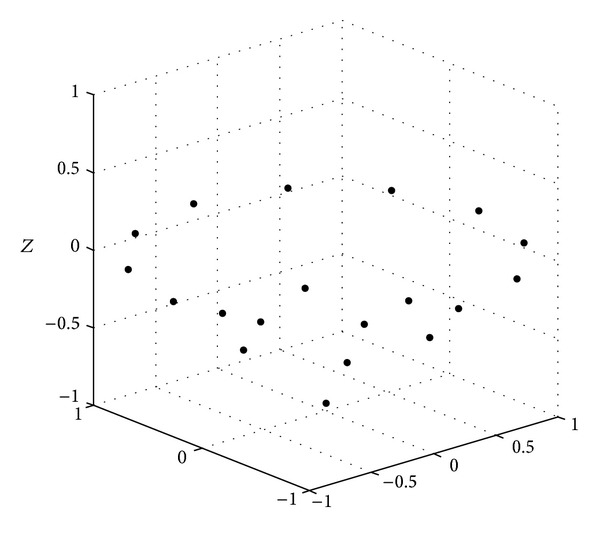
Element distribution of VRA for LEO satellites with concentric excitation.

Figures 6, 7, and 8 show the spatial element distribution for the optimization cases.  Table 1 presents the numerical values for the spatial distribution and excitations. As an important guideline, a smaller aperture in *z*-direction is also necessary for a bigger EOC; that is, a smaller aperture of the antenna array is required for a bigger isoflux curvature. In this case, the antenna array for a LEO satellite has a smaller aperture than the cases of GEO and MEO satellites. We could mention that in a case of a VRA at 2.8 Ghz, the wavelength is 10.71 cm; this would result in 8.16 cm, 4.05 cm, and 10.1 cm of apertures in *z*-direction for the concentric excitation of GEO, MEO, and LEO, respectively. It could be noted that the antenna arrays are not bulky. Finally, Table 2 presents a comparison of the numerical values for the obtained designs and the designs presented previously in the literature for the same application. It is shown that the VRA performs a better SLL with similar number of excitations. This is an attractive solution to provide a uniform terrestrial coverage on the earth and represents also an option to reduce the volume occupation of the hardware which is crucial in satellite applications.

## 5. Conclusions

This investigation reported a VRA for a satellite system with an accurate uniform terrestrial coverage by PSO. The geometry could provide an acceptable solution for reducing the volume occupation and power efficiency in the antenna system. The obtained design performs a better behavior in terms of accuracy of the isoflux shape with respect to the designs presented previously in the literature. Since the point of view of the crucial factor to simplify the beamforming network in any antenna array implemented in a satellite for isoflux radiation, the authors proposed an alternative geometry so that the levels of excitations are concentric and uniform. The main idea is to avoid a complicated heat dissipation system in a satellite as well as a complicated beam forming network that surely would add volume and weight to the satellite. This paper includes only the study of the array factor. However, the main intention of the work is to find a new geometry that permits a uniform coverage with consideration of the simplification of the beamforming. This study proposes a new alternative for this issue by showing theoretical results. Undoubtedly, this new geometry permits this end. It is worthy to mention that this fundamental study is the first stone of a more complex work to be developed in the future; the manufacturing of this type of array is still in develop with the problems associated with the type of antenna element, coupling effects, frequency band, feeding system, and so on.

## Figures and Tables

**Table 1:** Numerical values of antenna excitations and locations.

Vector	VRA for GEO		VRA for MEO		VRA for LEO	
	Uniform excitation	Concentric excitation	Uniform excitation	Concentric excitation	Uniform excitation	Concentric excitation
*Z* (meters/*λ*)	0.4411, 0.4094, 0.4057, 0.3520, 0	−0.7621, −0.7620, −0.6288, −0.6286, 0	0.4167, 0.4166, 0.4166, 0	−0.3782, −0.3685, −0.3109, 0	NA	0.9456, 0.4457, 0

*S* (meters/*λ*)	0.5000, 0.5000, 0.7316, 1.5000	0.5, 0.5, 1.2134, 0.5135	0.5513, 0.6510, 0.5000	0.5001, 0.6272, 0.8383	NA	0.6668, 0.5592

*L* (amplitudes)	1, 1, 1	−3.999, 0.9999, −0.6439	1, 1, 1	−3.5406, −0.2102, −1.0001	NA	−3.9983, −1.8402, −0.5333

**Table 2:** Comparison among the obtained designs and previous designs.

Design	Procedure	*N_T_*	SLL (dB)	Levels of excitations	Discrepancy of directivity between mask and pattern
VRA (GEO)	PSO	61	−27.5	3	|0.1 dB|
VRA (GEO)	PSO	61	−13.4	1	|0.1 dB|
VRA (MEO)	PSO	37	−31.0	3	|0.1 dB|
VRA (MEO)	PSO	37	−5.8	1	|0.4 dB|
VRA (LEO)	PSO	19	−7.4	3	|3 dB|
Reference [10] (GEO)	PSO	61	−19.0	3	|0.5 dB|
Reference [11] (MEO)	PSO	37	−19.3	3	|1 dB|
Reference [7] (GEO)	Deterministic	61	−15.0	12	Not specified
Reference [8] (MEO)	Deterministic	144	Not specified	Not specified	Not specified
Reference [8] (GEO)	Deterministic	100	Not specified	Not specified	Not specified
